# Supplementary material for: Disentangling neuronal inhibition and inhibitory pathways in the lateral habenula
Source: Sci Rep. 2020 May 22;10:8490. doi: 10.1038/s41598-020-65349-7 (PMC7244525; doi:10.1038/s41598-020-65349-7)
Supplement: Supplementary file 1 — Supplementary Figures. [file 41598_2020_65349_MOESM1_ESM.pdf]

Supplementary material

Disentangling neuronal inhibition and inhibitory pathways in the lateral habenula

Jack F. Webster[1], Rozan Vroman[1], Kira Balueva[2], Peer Wulff[2], Shuzo Sakata[1] and Christian Wozny[1,*]

[1] Strathclyde Institute of Pharmacy and Biomedical Sciences

University of Strathclyde

161 Cathedral Street

Glasgow

G4 0RE, UK

[2] Institute of Physiology

Christian-Albrechts-University of Kiel

Hermann-Rodewald-Straße 5

24118 Kiel, Germany

* Corresponding author: christian.wozny@strath.ac.uk

Phone: +44 (0)141 548 2122

Supplementary figures

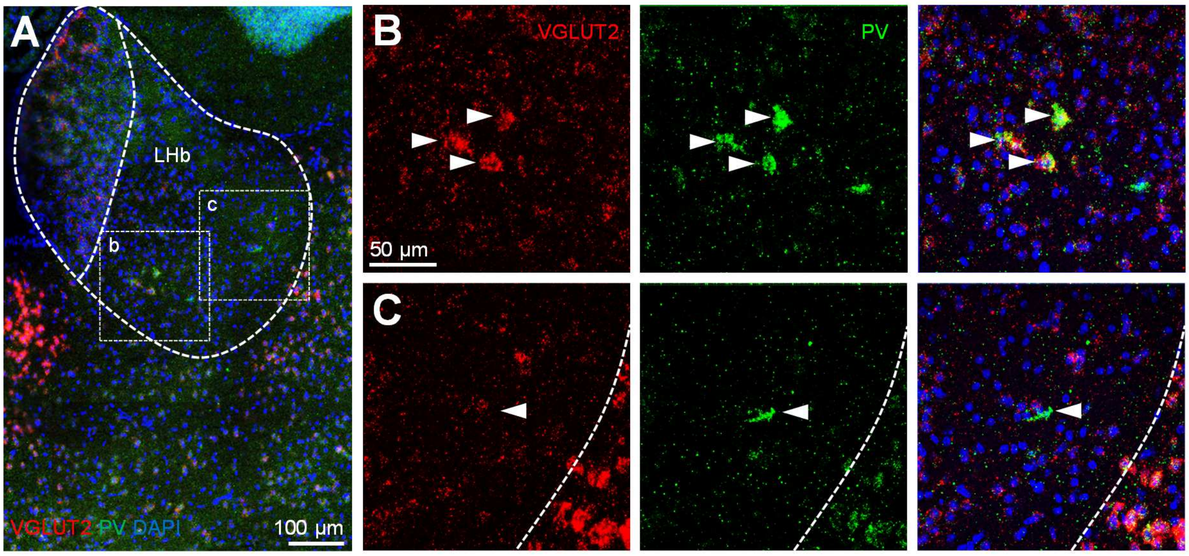

Supplementary Figure 1:

PV-positive neurons in the medial LHb are glutamatergic. (A) Overview image of the LHb from PV / VGLUT2 double in situ hybridisation. **(B)** Zoom of boxed region in (A) depicting PV / VGLUT2 double-positive neurons in the medial LHb. **(C)** Zoom of boxed region in (A) depicting PV-positive neurons which were negative for VGLUT2.

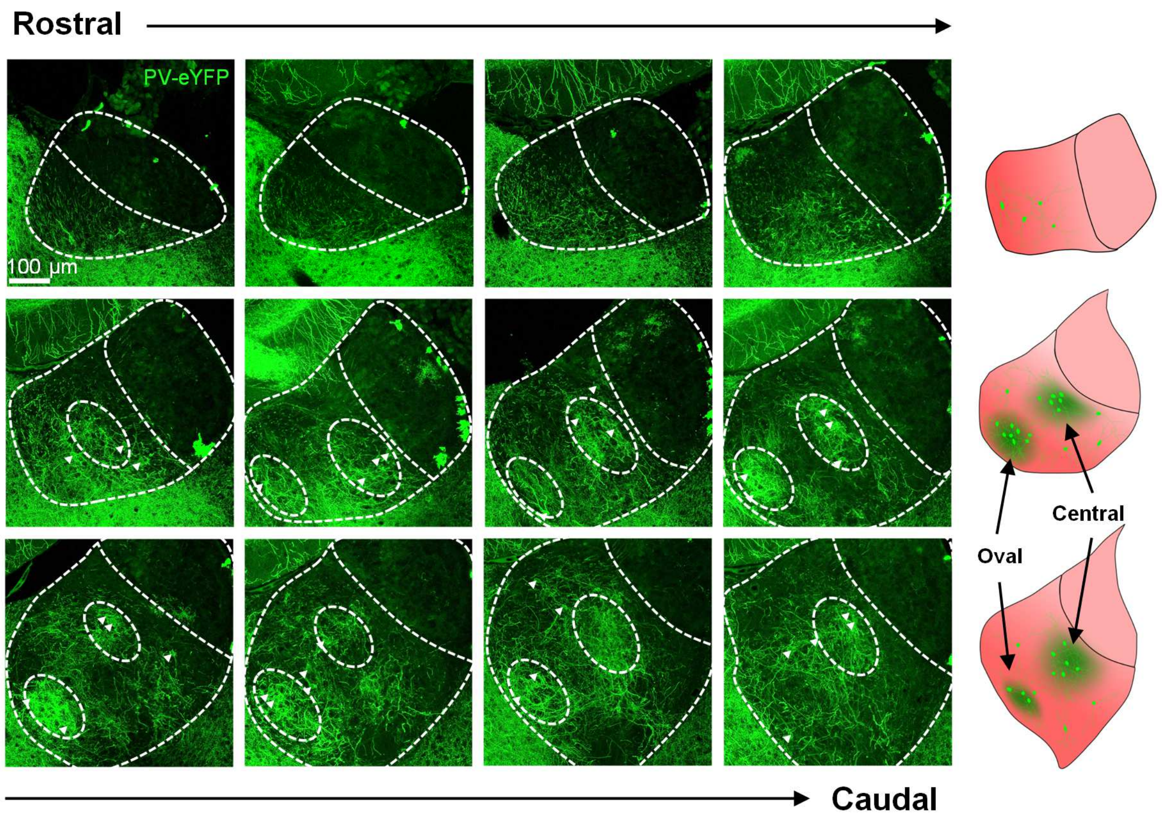

Supplementary Figure 2:

Localization of PV-positive neuronal somata and processes throughout the LHb. Left: Confocal micrographs of 30 µm thick maximum intensity projections of habenular sections from PV-IRES-Cre::Ai32 mice (N = 2) depicting localization of PV-positive neuronal somata and neuronal processes throughout the LHb in the rostral-caudal plane. 60 µm thick spacing between images. Arrowheads indicate PV-positive neurons. Right: graphical illustrations of habenular sections indicating the oval and central lateral habenular sub-regions, where PV-expression was most prominent. Images each represent one third of the habenula in the rostral-caudal plane.

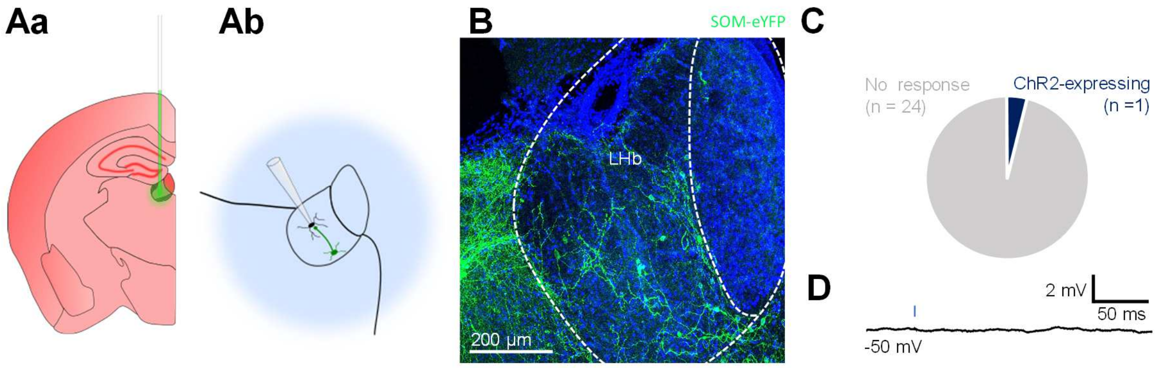

Supplementary Figure 3:

SOM-positive LHb neurons do not mediate local inhibition. (Aa) Schematic illustrating stereotaxic injection protocol of AAV9 into the LHb of SOM-IRES-Cre mice (N = 4). **(Ab)** Schematic illustrating electrophysiology recording protocol for LHb neurons following stereotaxic viral injection. Transduced SOM-positive neurons are photostimulated while recording from nearby LHb neurons. **(B)** Confocal micrograph depicting virally-transduced SOM-positive neurons within the LHb. **(C)** Pie chart quantifying fraction of neurons responsive to photostimulation. **(D)** Representative example trace from a neuron which showed no response to photostimulation. Trace is an average of multiple individual sweeps.

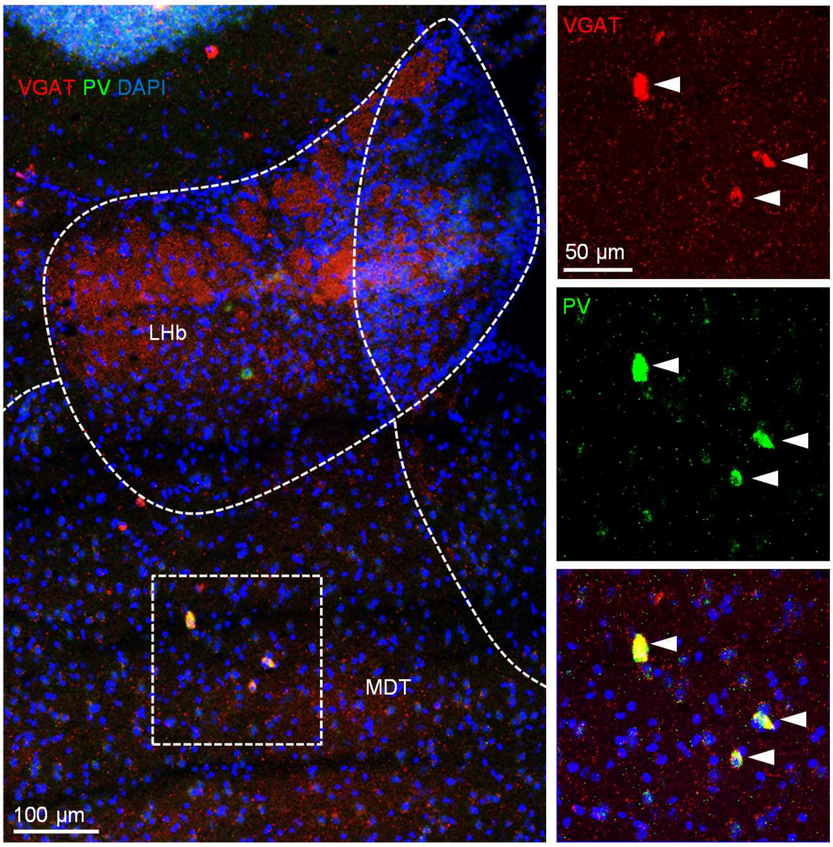

Supplementary Figure 4:

PV-positive neurons in the MDT are GABAergic. In situ hybridization depicting VGAT / PV double positive neurons in the MDT.
